# Depression as a Function of Social Support in Transgender and Cisgender Individuals with Sexually Transmitted Diseases

**DOI:** 10.3390/ijerph18052462

**Published:** 2021-03-03

**Authors:** Tahira Yousuf, Mahwish Naz, Candace B. Roberson, Suzanna M. Wise, David L. Rowland

**Affiliations:** 1 Institute of Professional Psychology, Bahria University, Karachi 75260, Pakistan; tahiraysf@yahoo.com (T.Y.); mahwishnaz1092@gmail.com (M.N.); 2 Department of Psychology, Valparaiso University, Valparaiso, IN 46383, USA; candace.roberson@valpo.edu (C.B.R.); suzy.wise@valpo.edu (S.M.W.)

**Keywords:** transgender, cisgender, depression, social support, self-esteem, STI, Hepatitis C, HIV

## Abstract

This study focused on the relationships among social support, self-esteem, and depression in transgender and cisgender individuals suffering from an incurable or curable sexually transmitted disease. Data were collected from 210 participants with an STI using a semi-structured interview along with culturally adapted standardized instruments. Results indicated no differences between transgender and cisgender groups in depression, although there were large differences in social support and self-esteem. Preliminary regression analysis identified only STI type and duration of STI as significant predictors of depression. However, when moderating roles for both social support and self-esteem were tested, each added to the explained variance and, equally importantly, revealed the effects of both gender status and social support on depression. These findings not only demonstrate how the compound stressors of gender minority status and STI type affect depressive symptoms, but also reveal the critical role that social support can play in mitigating depressive symptoms in those with gender minority status. Findings are interpreted within the context of South/Central Asian cultures that have pre- and post-colonial traditions regarding the social role of non-binary individuals.

## 1. Introduction

Sexually transmitted infections (STIs) represent an escalating public health issue in many parts of the world, despite probable underreporting in countries with low STI surveillance practices [1,2]. World Health Organization (WHO) statistics indicate that 499 million adolescents and adults (ages 15–49) were newly infected with curable forms of STIs across the world in 2008, a mix of chlamydia, syphilis, trichomoniasis, and gonorrhea [3]. This pattern is not just characteristic of emerging or developing nations. In the USA, underfunded health departments struggle to manage increasing sexually transmitted diseases: in 2018, the U.S. reported 115,000 cases of syphilis and 580,000 cases of gonorrhea—the highest number in nearly three decades. The highest number of chlamydia cases was also reported at 1.7 million [4].

Some members of the population are more vulnerable to STIs than others. For example, human papillomavirus (HPV), a disease leading to cancer, infects 290 million women at any given time [3,5,6]. Furthermore, the Centers for Disease Control and Prevention (CDC-USA) have acknowledged that, as high as these numbers are, STIs frequently go undiagnosed and thus are underreported, even in countries with high economic development. Not only do women with undiagnosed, untreated, recurring, or chronic STIs risk serious reproductive damage, but those who do eventually seek treatment often bear further burden by assuming responsibility for a treatment-reluctant partner [6].

Members of sexual and gender minority groups are among those with the highest risk for an STI, including transgender and cisgender men who have sex with men (MSM), transgender women, and genderqueer non-binary people [7,8,9,10]. Specifically, the most widespread STI transmitted through sexual interaction is HIV, occurring not just in emerging nations but within nearly every society in the world [11]. Although HIV occurs in people of all sexualities and genders, it disproportionately affects sexual minority men and transgender people. Gonorrhea shows similar trends, with “exploding” rates occurring within bisexual, gay, ethnic, and indigenous communities throughout the world [12,13,14].

Unfortunately, gender and sexual minority individuals experience multiple barriers to accessing health care and seeking treatment, having not only to overcome heterosexism and discrimination at individual, group, and institutional levels, but also to deal with oft-reported negative medical experiences when seeking treatment for sensitive gender-related and reproductive health (e.g., STI) issues. According to the Minority Stress Model [15], the understandably avoidant behavior that results from this negative or potentially negative experience places minority individuals at further risk, perpetuating a cycle of inadequate care, deteriorating health, and diminished sense of well-being [7,16].

### 1.1. Psychological Effects of Sexual/Gender Minority Status and STI Status

When gender and sexual minority individuals lose their primary support systems, they are pushed to the margins of society to seek housing, support, resources, and employment. Multiple stressors may negatively impact their ability to cope; as a result, their health-protective behaviors diminish, and health-averse behaviors increase. They become at risk for further victimization and exploitation, especially the younger and more under-resourced they are [17]. Due to the commonplace experiences of perceived and internalized stigma, isolation, discrimination, and victimization, this patient cohort shows disproportionately high rates of problems, up to 60% in some communities, commonly manifesting as depression, anxiety, substance abuse, self-injury, suicidal ideation, and sometimes death [1,18,19,20,21].

At the same time, STI status is independently associated with stigmatization, as well as greater likelihood of risky behaviors, low quality of life, poorer reproductive and physical health, and lack of motivation for change [22,23]. In addition, STI-positive status imparts its own risk for mental health issues, in particular, low self-esteem and depression [1,24,25,26]. Thus, having an STI and identifying as a sexual and/or gender minority represent independent risk factors for depression. Yet, the compounding effects of such risks on the psychological health of this population have not received adequate attention, particularly within cultures where acceptance toward sexual and gender diversity is limited [27].

### 1.2. The Role of Social Support

Often, the coming out process for someone who identifies as other than cisgender or heterosexual results in family-of-origin rejection, societal discrimination, and reduced opportunities for upwardly mobile education and employment. In contrast, when LBGTQ individuals are supported in the coming out process by their families and can continue their natural developmental processes without persistent fear of retaliation and discrimination, they are mentally, physically, and emotionally healthier, as well as more resilient [28]. As noted by Kosciw et al., “Outness was related to higher victimization but also to higher self-esteem and lower depression” ([28], p. 167), two of the mental health variables explored in this present research.

For those sexual minority individuals who find themselves socially marginalized or rejected (for whatever reason), the negative impact on psychological well-being can be mitigated through adaptive coping strategies and resilience [29]. One key factor that helps protect against negative psychological effects is an individual’s perceived level of social support [30]; thus, persons may cope with marginalization and isolation by seeking individual and community support for stability, safety, companionship, and livelihood [31]. Indeed, evidence indicates that depression associated with either transgender and/or STI status can be mitigated in part by high levels of social support [18,30]. Despite this general association between perceived social support and (lower) depression, greater understanding of the role of social support in individuals with STIs is warranted [1,11], particularly when those individuals are members of a sexual or gender minority group.

### 1.3. Situating Risk Factors for Psychological Effects within Specific Cultural Milieus and Historical Contexts

Research on the psychological effects of sexual and/or gender minority status in non-Western nations/cultures has emerged in recent decades as an interdisciplinary topic of interest, though such initiatives have been limited both in number and in scope (e.g., geo-cultural regions). Yet, a person’s psychological response to the distress of stigmatization is greatly influenced by the cultural values and traditions in which the individual is embedded. That is, culture defines acceptability and marginalization of various socio-sexual roles and therefore plays a critical role in the way men and women describe, interpret, and ascribe meaning to their specific status [9,32,33,34]. Indeed, the interpretation/meaning-making process within a socio-cultural tradition is often as important for the individual as the actual symptoms of the disease (STI) or the person’s social location (gender and/or sexual identity). Yet most attempts at understanding the psychological experiences of men and women to negative situations/conditions have relied on studies conducted in educated and (sexually) more open Western-oriented samples, even though studies suggest that such samples may be among the least representative in the world [35].

In Pakistan, a Central Asian country with the second-largest Muslim-majority population in the world, marginalization due to gender minority and STI status places heavy psychological burdens on the individual. Transgender people in this geo-cultural region face significant human rights issues [36], and despite their relatively limited numbers in Pakistan, they disproportionally account for 17.5% of HIV cases [37]. The term *transgender* in Pakistan reflects a mix of identity labels within a broad community of socially marginalized individuals [38] of varying sexual and gender minority statuses. While this term is typically found in Western contexts, it is increasingly applied across the world [39]. Specifically, in South Asian countries such as India, Pakistan, and Bangladesh, *transgender* includes many labels which describe sexual orientation, biological sex, and gender identity, such as, “eunuchs, transvestites, homosexuals, bisexuals, hermaphrodites, androgynous, transsexuals, and gynemimetic; beyond this, they are also referred to as people who are intersexed, emasculated, impotent, transgendered, castrated, effeminate or somehow sexually anomalous or dysfunctional” [40]. In Pakistan, these labels may be organized under the umbrella gender identity label, *khwaja sira*, which in turn encompasses the terms *zennana*, *hijra*, and *khunsa*.

Pakistan and neighboring countries (e.g., India, Bangladesh, Nepal) afford a distinctive opportunity for understanding the relationships among gender minority distress, social support, and depression—here, religious and cultural ethics have played an important role in the social contract with transgender individuals, one that dates back to pre-colonial times. Prior to British rule, transgender people (*hijra*) had an important role and status, where during the Mughal rule they guarded the ladies of the harem and were considered servants of the nobility, often being promoted to key advisory roles [41]. In the historical tradition of the region, they were attributed significant value due to the belief that they blessed people with fertility, having been granted their power by the goddess Bahuchara Mata herself [42]. Against this tradition, however, in 1870, the British colonized Central Asia and passed morality laws such as the Criminal Tribes Act of 1871 and the Dramatic Performance Act of 1876 which labeled the hijras as “sodomites” and restricted their activities, inheritance, and other rights [42]. Thus, in the post-colonial era of today, members of sexual/gender minority groups in Pakistan experience social stigmatization and marginalization. On the other hand, giving a nod to its pre-colonial past, Pakistan’s Supreme Court has recently (2018) provided official recognition to the “third” gender in the citizen registration category, a recognition that affords both voting rights and access to health care [37,39].

The effects of stigma, rejection, and isolation by families of origin tend to motivate individuals to seek supportive networks that represent the chance for safety, security, and growth-oriented opportunity [31]. Accordingly, transgender people often form their own strong social support systems, “transgender communities,” found in many countries worldwide, including Pakistan [43,44,45]. These communities confer benefits on their members, including mitigating the effects of stigmatization and loneliness, providing social support through opportunity for friendships and relationships, and serving as a surrogate family [46,47,48,49,50,51,52]. In Pakistan, such communities, or clans, are sometimes structured as a master–disciple (*guru*–*chela*) hierarchy, where older members mentor new members and provide a pathway to earning a living through dancing, begging, or blessing [32]. Although transgender individuals who live with their elders in such communities have reported higher levels of resilience and self-esteem, these communities are located in ghettos, where members lack equitable social benefits relative to cisgender peers; as a result, long-term health and longevity typically suffer [38].

### 1.4. Rationale and Aims

The psychological health of transgender people has largely been ignored in many developing countries, where limited health care resources must often focus on managing and controlling highly infectious diseases. Even less understood is how health risks such as STIs might compound the psychological burden on transgender individuals, and the extent to which social support systems might mitigate this effect [18]. In this comprehensive analysis, we explored the role of social support and self-esteem on psychological depression in groups of transgender and cisgender individuals suffering from either a curable or incurable STI. As part of the analysis, we also explored the role of several covariates, including the duration of the STI, marital status, and the presence of other major disease states. Understanding the relationships among such variables could inform the development of strategies to improve the psychological well-being of individuals suffering from STIs in both the transgender and cisgender communities.

Specifically, in this study we: (1) assessed and compared depression levels across groups of transgender people and cisgender men and women in Pakistan with either a curable or incurable STI; (2) identified factors that predict depression in these two groups, including the levels of social support and self-esteem, and several socio-demographic variables; and (3) explored a potential role for social support and self-esteem as moderating variables in explaining differences in depression between transgender and cisgender groups.

## 2. Methodology

### 2.1. Participants

A total of 210 individuals from both private and government sector organizations in Karachi, Pakistan, were recruited through quota and convenience sampling. Of these, 95 self-identified as transgender and 115 self-identified as cisgender, with an age range of 17 to 39 years. In the present study, the term *cisgender* is used to denote individuals whose assigned sex at birth matches their current gender identity whereas, as noted previously, the term *transgender* in Pakistan reflects a mix of identity labels within a broad community of socially marginalized individuals [38] of varying sexual and gender minority statuses. Given the complex make-up of the transgender community in terms of sexual identities, as well as the reluctance of cisgender individuals to reveal their sexual orientation, distinctions of these more specific gender identities or sexual orientation identities were not sought from participants, with participants in this study simply referred to as either *cisgender* or *transgender*.

All participants had been diagnosed with a verified sexually transmitted disease of either HIV or Hepatitis C (HEP-C) at least six months prior, enabling comparison between an incurable and curable illness STI [53]. HEP-C may be transmitted through various means, including sexual contact. Approximately 25% of HEP-C cases clear on their own; the remaining cases typically require 8–24 weeks, with a typical treatment regimen involving 12 weeks.

Socio-economic status of participants was determined by household income and expenditure indexed to information provided by the Federal Bureau of Statistics [54]. Characteristics of the overall sample, as well as for the transgender and cisgender groups, are presented in Table 1.

### 2.2. Measures

The interview and measures were presented in Urdu, the national language of Pakistan, so participants could understand and respond more easily. The following forms and assessment instruments were used:

Informed Consent: Prior to data collection, the informed consent form was completed and signed by participants. This document described the details of this study, specified conditions for giving informed consent, and explained that participants could withdraw from the study for any reason and at any time without consequence. Participants were assured that their information would remain confidential and private, and that their identity would not be associated with this study or data in any way.

Semi-Structured Interview Form: Each participant completed a semi-structured interview that gathered information regarding their self-identified gender (transgender or cisgender), age, education, marital status, socio-economic class, birth order, number of siblings, family structure, nature of sexually transmitted illness, duration of STI, hospital/organization, and a short medical history that included other major lifetime illnesses.

Siddiqui Shah Depression Scale (SSDS): Depression was assessed with the SSDS [55]. This Urdu language diagnostic represents a culturally adapted assessment instrument that considers not only local norms and values but also the local lexical categories for emotion [56]. The SSDS is comprised of 36 items, with each evaluated on a 4-point scale, where “0” denoted no sadness, “1” normal sadness, “2” mild depression, and “3” severe depression, generating an overall index. Scores between 26 and 36 represented “mild depression,” 37–49 “moderate depression,” and 50 and above “severe depression.” Test-retest reliability is 0.85, with discriminant validity of 0.81, and construct and concurrent validity of 0.64 [55], not unexpected due to the cultural-adaptation.

Social Support Scale (SSS): This Urdu version instrument measures social support [57] characterized by physical and psychological comfort provided by others around the person, for example, one’s friends and family members. It consists of 52 items assessed on 4-point scales in five different areas—informational support, tangible aid, emotional support, esteem support, and social network support. Most often, the test generates a single composite score, although in some instances the esteem support scale is calculated as a separate subscore. Split-half reliability has been reported at r = 0.79.

### 2.3. Procedure

This study was reviewed and approved by the Ethics Committee of Bahria University, Institute of Professional Psychology, Karachi, Pakistan. Recruitment for both the transgender and cisgender groups involved a multi-step process. A letter of permission describing details of the research project (including copies of the questionnaires) was provided to the authorities of selected hospitals and organizations in Karachi, Pakistan, including Jinnah Postgraduate Medical College, Civil Hospital, Sindh Government Hospital, Pakistan Society for HIV Patients & Sindh AIDS Control Program, and various NGOs, including ones specifically associated with the transgender community. Medical and administrative staff working with these patients were approached for permission to study patients’ medical records prior to contacting individuals for this study.

After careful review of patients’ files, those who had been diagnosed positive for sexually transmitted HIV or Hepatitis C (HEP-C) were contacted for further study and possible participation, which was explained to them as completely voluntary. Although initially many candidates were reluctant to participate due not only to their serious medical status but also to their fear of being “outed,” reassurance of confidentiality and the potential value of the research, along with empathy, building trust, providing guidance, and unconditional positive regard by one of the researchers (TJ) allayed the fears of many candidates who subsequently expressed their interest in participating.

Although Urdu is one of the national languages of Pakistan, some individuals converse primarily in their regional/provincial languages and therefore may have needed assistance with the lexicon of the assessment instruments which was provided with the translation aid of the researcher.

### 2.4. Analytical Strategy

We explored differences in depression, social support, and self-esteem through a two-factor analysis with gender identity status (transgender vs. cisgender) and STI type (HIV/HEP-C) as independent variables, including age and education as covariates as these differed across gender groups. Because neither covariate was significant, both were dropped from further analyses. For regression, preliminary analyses were conducted to determine correlations among potential demographic covariates in order to eliminate one of each pair of collinear variables. Having a drug addiction was associated with greater depression, but because it also showed associations with other covariates of interest, including type of STI, level of social support, and level of self-esteem, it was included in the initial exploratory regression model but (due to its non-significance) not included in the final model. In addition, being married was moderately associated with greater social support and greater self-esteem, so this variable also was dropped as a potential covariate in the final regression model.

## 3. Results

### 3.1. Description of Transgender and Cisgender Groups

Transgender participants were slightly older and less educated, and more had non-STI chronic illnesses (but less drug addiction) than cisgender participants. On other variables, the groups were similar (Table 1).

### 3.2. Comparisons of Outcome Variables across Transgender and Cisgender Participants and STI Types

There were no overall differences in depression between the two gender groups, but both STI and the interaction of STI by gender group were significant. Specifically, those with HIV showed greater depression, with the interaction indicating that transgender participants with HEP-C showed greater depression than cisgender participants with HEP-C, but no differences in depression occurred between transgender and cisgender participants with HIV (Table 2).

Social support was higher in the transgender group, and those with HEP-C reported significantly higher social support than those with HIV. No STI by gender group interaction was found.

Self-esteem was lower in the transgender group and in those with HIV. In addition, the interaction of STI by gender group was significant, reflecting the pattern that transgender participants showed low self-esteem independent of their STI, whereas cisgender participants with HIV showed lower self-esteem than those with HEP-C (Table 2).

### 3.3. Predictive Factors for Depression Using Regression Analysis

In regression analysis with depression as the outcome variable, we included the following predictor variables: group membership (transgender vs. cisgender), social support, self-esteem, type of STI, and duration of STI. The adjusted R^2^ value of 0.22 was significant (*p* < 0.001), with both type and duration of illness being significant covariates (Table 3, Model 1). As suggested by the previous analyses, those with HIV showed greater depression than those with HEP-C (*p* < 0.001), and those with shorter duration of the illness showed higher depression (*p* = 0.018).

### 3.4. Moderating Effects of Social Support and Self-Esteem on Depression

The findings that there were differences in social support and self-esteem between transgender and cisgender groups, but that neither of these variables significantly predicted depression in the regression model, suggested that these variables might moderate the differences in depression between transgender and cisgender participants through their interaction effects. Testing for moderating effects involves a two-step regression procedure in which a first model is run without the interaction term, then the second model is run with the interaction term included to determine whether the change in explained variance (R^2^) is significant [58].

For social support, the change in R^2^ was significant (*p* = 0.012), with explained variance increasing by 2.4% (Table 3, Model 2). For self-esteem, the change in R^2^ was also significant (*p* = 0.048), with explained variance increasing by 1.5% (Table 3, Model 3). When both interaction terms were included, the overall increase in R^2^ was 3.7% (*p* = 0.007) (Table 3, Model 4).

Furthermore, in the final model (Model 4), two covariates that previously exhibited no predictive power for depression—group membership and social support—emerged as significant, with depression being related to transgender status and lower social support. These results emphasize that in order to understand the role of social support and/or self-esteem on depression, the participant’s identification as transgender or cisgender is important.

## 4. Discussion

This study—one of only a few of its kind—has provided insight into the role of social support on depression in transgender and cisgender individuals with either a curable or incurable STI in a non-Western though regionally distinctive setting [42,59,60,61]. The negative impact of having an STI—curable or incurable—on both self-esteem and depression in this Pakistani sample is consistent with prior research on this topic [24,25]. Not surprisingly, *type* of STI also played a major role in all three outcome variables: HIV patients showed greater depression, lower perception of social support, and lower self-esteem than HEP-C patients, findings also consistent with the previously documented diminished sense of hope and self-worth for individuals suffering from an incurable STI [62,63].

Differences also emerged between trans and cisgender groups: the transgender group reported lower self-esteem *but* higher perceived social support. Quite unexpectedly, the main outcome variable—depressive symptoms—did *not* differ between trans and cisgender groups, a finding that is better understood when the combined effects of marginalized gender status and STI status are considered, and when interaction effects are examined.

### 4.1. The Combined Effects of STI and Marginalized Gender Status

Broadly speaking, participants who were both transgender and HIV positive—that is, with the dual stressors of gender minority status and an incurable STI—reported the highest levels of depression. In contrast, cisgender participants with HEP-C—those with the lowest stressors—not only reported the lowest levels of depression but also the highest levels of self-esteem.

More detailed inspection indicated that participants with HIV—whether trans or cisgender—reported high levels of depression, with both groups falling into the “severe” depression category. Participants with HEP-C—both trans and cisgender—reported comparatively lower levels of depression, with cisgender HEP-C participants reporting the least depression. In other words, depressive symptoms in the transgender group were less affected by STI type than depressive symptoms in the cisgender group. These findings suggest that (low) psychological well-being of transgender individuals was linked much more to their gender minority status than to their STI type (curable or incurable).

Self-esteem exhibited a somewhat related pattern. Transgender groups—independent of their STI status—showed equally low self-esteem, indicating once again that the salient factor for self-esteem in this group was their gender minority status (and not STI type). In contrast, cisgender HIV participants showed just as low self-esteem as transgender participants, but cisgender HEP-C participants showed much higher self-esteem. In sum, STI type was much more relevant to predicting depressive symptoms and self-esteem in cisgender participants than in transgender participants.

### 4.2. The Moderating (Interactive) Roles of Social Support and Self-Esteem

The apparent lack of difference in depression between trans and cisgender groups in our study contrasts with the research literature that predicts greater depression in the transgender group [16,19,20]. Understanding this result requires consideration of the moderating roles that social support and self-esteem played in the cisgender vs. transgender groups. Specifically, the significantly greater social support felt by transgender participants helps explain why they were *not more depressed* than cisgender counterparts. That is, strong social support appears to mitigate depressive symptoms that occur in response to marginalization from gender minority status. Furthermore, when the moderating effect of self-esteem was added to that of social support, an additional 3.7% of variance was explained in the model (An r-squared value of 3.7% is comparable to f-squared (Cohen’s d) of approximately 4.0%, considered a respectable size for a moderating effect (see http://davidakenny.net/cm/moderation.htm#, accessed on 3 March 2021).

Notably, when the moderating effects of social support and self-esteem were accounted for, differences in social support and gender group membership emerged as significant predictors of depression. Specifically, social support was associated with lower depression, whereas transgender identity was associated with greater depression, effects that were masked until group interaction variances were controlled. In this respect, our study has uniquely demonstrated the heavy psychological burden borne by transgender individuals who have an incurable STI [1,24,25], an effect that bears on the individual’s self-esteem but also one that can be countered in part by a system of strong social support.

### 4.3. Interpretation and Implications

Individuals who identify as transgender represent a highly diverse group of gender identity and sexual identity labels in many parts of the world. For some, there is both pride and resilience in these identities, and historically, some cultures have honored and revered transgender individuals for their spiritual gifts. Nevertheless, the colonial and post-colonial landscape in South and Central Asia has created a system of societal stigma that relegates transgender people to the margins, resulting in family rejection, societal and institutional discrimination, and a life often characterized by beggary. Such conditions can have deleterious effects on the mental and physical health of individuals in these populations [64].

As in many cultures and countries, marginalized individuals typically attempt to find sources/locations of support, safety, and companionship; and such is the case for gender minority individuals in Pakistan. Approximately 10,000 transgender individuals live in Pakistan, with most residing in collective transgender clans led by gurus [42], fostering a sense of “transgender community belongingness” [59] through both identity and locale. Although guru–chela organizational structures can be exploitive and abusive [42], most transgender people perceive the *hijra or khwaja sira* community structure to be a vital source of protection against wide-ranging forms of discrimination in society, and they feel grateful to their gurus for creating this space.

As suggested by our study, these “transgender communities” undoubtedly serve an important role in the psychological health of their members, particularly those suffering from stigma arising from either minority status or STI-positive status. In fact, it is evident from our results that gender minority marginalization surpasses STI status as the predominant factor related to depression in transgender individuals. In contrast, although the type of STI—whether curable or incurable—played a role in the level of depressive symptoms in both cis and transgender individuals, its overall role on psychological well-being was much greater in cisgender participants.

Along with transgender support communities, NGOs are playing an increasingly important role in the support of reproductive health in many emerging nations, including the health of sexual and gender minorities. NGO organizations in Pakistan not only aid in the stabilization and safety efforts essential to treatment and prevention of STIs, but they also provide opportunities for employment training, education, and health care access for transgender people [32]. Furthermore, recent legal changes in several Central/South Asian countries (e.g., Bangladesh, Pakistan, India, and Nepal) have led to official recognition of the “third gender,” placing this population on a more equal footing regarding access to social resources, education, and employment. It will, however, take time to determine whether and how this new legal status improves the lives of transgender people in this geographic region [65].

The implications for improving the psychological health of both trans and cisgender individuals suffering from an STI are clear from the present study. Ensuring adequate support systems, no matter the source, is critically important to psychological well-being. For example, supportive cognitive-behavioral interventions could help transgender individuals develop positive stress management skills for controlling anxiety, depressive symptoms, and negativity. At the community level, NGO-based human service professionals might attend to interpersonal dynamics and organizational structures within transgender communities so as to maximize members’ feelings of social support [66]. In addition, these professionals, in building trust with the transgender communities, could assist community members in accessing needed services (housing, health clinics) and could serve as experts/advocates to the outside world in matters related to policy, law, and human rights.

### 4.4. Limitations

A major limitation of this study was its cross-sectional nature, thereby preventing causal interpretations among gender status, STI type, social support, and depression. Future research that implements intervention strategies, at both the individual and transgender community levels could help establish needed cause–effect relationships, while concomitantly offering valuable mental health services to transgender individuals and communities.

A second major limitation to a nuanced data analysis was the lack of information regarding the sexual orientation and/or more specific gender identity types, given that specific identities likely represent added stressors. Due to the stigmatization of difference in Pakistani society, the information provided by participants on these points would likely not be reliable. For instance, cisgender individuals might generally be unwilling to answer this question or even participate in this study if they were asked to do so. This knowledge gap, however, is not specific to this geo-cultural region. Even in the USA where diversity data are typically more readily available, the CDC does not provide specific STI screening guidelines for transgender people but instead relies on sex-specific recommendations, thus leaving out important areas of inquiry in sexual behaviors, exposures, and outcomes in high-risk populations [2]. Including such information in STI surveillance reporting could provide more accurate information about disproportionate infection rates [2]. Additionally, as long as the collection of such data does not raise further barriers to STI treatment, implementation of such a strategy would enable more targeted interventions and education campaigns.

Our study also did not specify the source of social support for participants, and although we had assumed that for transgender individuals it was derived mainly from membership in transgender communities, we also found that being married correlated moderately with participants’ perceived levels of social support. Other sources, including economic and family resources, may have also played important though different roles for transgender and cisgender participants.

Finally, our results may be limited to transgender individuals in like circumstances, that is, those with a positive STI status. Individuals with access to resources to overcome the typical obstacles resulting from stigma (e.g., gainful employment), or those whose lifestyles minimize the risk for STI, might not show similar relationships as found in our trans and cisgender samples. Future research might include a wider age range of participants to test age cohort effects, specify sources of social support, indicate sexual orientation, identify other life stressors, and consider qualitative components that might reveal nuances undetected by quantitative assessment instruments.

## 5. Conclusions

Although depression levels across groups of transgender people and cisgender men and women were initially non-significant, both STI type and gender minority status predicted depression levels when the moderating effects of social support and self-esteem were considered. Specifically, social support was associated with lower depression, whereas transgender identity was associated with greater depression. Furthermore, gender minority marginalization surpassed STI status as the predominant factor related to depression in transgender individuals. In contrast, although the type of STI—whether curable or incurable—played a role in the level of depressive symptoms in both cis and transgender individuals, its overall role on psychological well-being was much greater in cisgender participants.

## Figures and Tables

**Table 1:** Demographic and medical characteristics of participants.

Gender Status	Transgender (*n* = 95)	Cisgender (*n* = 115)	Significance
Characteristic	*f (%) or mean (SE)*	*f (%) or mean (SE)*	*p*-Value
**Age**	30.3 (0.47)	29.0 (0.49)	0.051
**Education**			
Non-Literate	69 (72.6)	53 (46.3)	
Primary/Middle	14 (14.7)	33 (28.7)	0.002
Intermediate	11 (11.6)	27 (23.5)	
College	01 (01.1)	02 (01.7)	
**Income Group**			
Lower	61 (64.2)	70 (60.9)	
Middle	32 (33.7)	40 (3482)	0.265
Upper	02 (02.1)	01 (09.0)	
Unknown	00 (00.0)	04 (03.5)	
**No. Siblings**	6.41 (0.19)	6.70 (0.22)	0.322
**Birth Order**	2.80 (0.18)	2.86 (0.18)	0.812
**Type of STI**			
HIV	55 (57.9)	71 (61.7)	
Hepatitis C	40 (42.1)	44 (38.3)	0.575
**Duration of STI (yrs)**	2.70 (0.14)	2.86 (0.22)	0.540
**Recruitment Source**			
Jinnah Postgrad Med Ctr	20 (21.1)	31 (27.0)	
Sindh Govt. Hosp	11 (11.6)	12 (10.4)	0.013
Pakistan Society	55 (57.9)	71 (61.7)	
Light House NGO	09 (09.5)	00 (00.0)	
**Chronic Illness**			
None	52 (54.7)	65 (56.5)	
Drug Addiction	25 (26.3)	41 (35.7)	0.039
Other	18 (18.9)	09 (07.8)	

Note: f = frequency, % = percent, M = mean, and SE = standard error. Comparisons were made with the *t*-test for means and chi square or Fisher’s test for frequencies.

**Table 2:** Major outcome variables across transgender and cisgender participants with either HIV or HEP-C.

Outcome Measure	Transgender (*n* = 95)		Cisgender (*n* = 115)		Effects (*p*-Value)		
	HIV (*n* = 55)	HEP-C (*n* = 40)	HIV (*n* = 71)	HEP-C (*n* = 44)	Gender	STI	Gender × STI
	Mean (SE)	Mean (SE)	Mean (SE)	Mean (SE)			
Depression	77.1 (3.0)	62.6 (2.2)	76.8 (2.5)	51.3 (3.4)	0.196	**<0.001**	**0.024**
Social Support	44.6 (3.3)	81.3 (7.0)	29.9 (1.6)	58.6 (6.8)	**<0.001**	**<0.001**	0.351
Self-Esteem	5.6 (0.2)	5.1 (0.3)	5.2 (0.6)	9.9 (1.0)	**0.002**	**0.001**	**<0.001**

Note: Significant *p*-values are indicated in bold.

**Table 3:** Regression results for predicting depression.

	Depression Model 1	Depression Model 2	Depression Model 3	Depression Model 4
Predictor	t (*p*-Value)	t (*p*-Value)	t (*p*-Value)	t (*p*-Value)
Intercept	14.83 (<0.001)	12.67 (<0.001)	10.66 (<0.001)	10.68 (<0.001)
Trans vs. Cis	1.10 (0.273)	−1.36 (0.176)	−1.39 (0.166)	−2.35 (**0.020**)
Social support	0.92 (0.359)	−2.80 (0.038)	0.90 (0.372)	−2.03 (**0.044**)
Self-esteem	−0.11 (0.912)	0.42 (0.675)	−1.97 (0.050)	−1.76 (0.079)
Type of STI	−6.02 (**<0.001**)	−6.19 (**<0.001**)	−5.66 (**<0.001**)	−5.83 (**<0.001**)
Duration of STI	−2.84 (**0.005**)	−2.80 (**0.006**)	−3.08 (**0.002**)	−3.03 (**0.003**)
Addiction	1.04 (0.299)	*	*	
Support by gender	*	2.55 (**0.012**)	*	2.48 (**0.014**)
Esteem by gender	*	*	1.99 (**0.048**)	1.91 (**0.055**)
R value	0.479 (**<0.001**)			
R-squared	0.225	0.249 (**<0.001**)	0.240 (**<0.001**)	0.263 (**<0.001**)
Change in R-squared		0.024 (**0.012**)	0.015 (**0.048**)	0.037 (**0.007**)

Note: Significant *p*-values are indicated in bold. * = Not Applicable.

## Data Availability

Data can be made available upon reasonable request.
